# Plasma Neutrophil Elastase and Elafin Imbalance Is Associated with Acute Respiratory Distress Syndrome (ARDS) Development

**DOI:** 10.1371/journal.pone.0004380

**Published:** 2009-02-06

**Authors:** Zhaoxi Wang, Feng Chen, Rihong Zhai, Lingsong Zhang, Li Su, Xihong Lin, Taylor Thompson, David C. Christiani

**Affiliations:** 1 Department of Environmental Health, Harvard School of Public Health, Boston, Massachusetts, United States of America; 2 Department of Biostatistics, Harvard School of Public Health, Boston, Massachusetts, United States of America; 3 Pulmonary and Critical Care Unit, Massachusetts General Hospital, Boston, Massachusetts, United States of America; 4 Department of Medicine, Harvard Medical School, Boston, Massachusetts, United States of America; University of Giessen Lung Center, Germany

## Abstract

**Background:**

We conducted an exploratory study of genome-wide gene expression in whole blood and found that the expression of neutrophil elastase inhibitor (PI3, elafin) was down-regulated during the early phase of ARDS. Further analyses of plasma PI3 levels revealed a rapid decrease during early ARDS development. PI3 and secretory leukocyte proteinase inhibitor (SLPI) are important low-molecular-weight proteinase inhibitors produced locally at neutrophil infiltration site in the lung. In this study, we tested the hypothesis that an imbalance between neutrophil elastase (HNE) and its inhibitors in blood is related to the development of ARDS.

**Methodology/Principal Findings:**

PI3, SLPI, and HNE were measured in plasma samples collected from 148 ARDS patients and 63 critical ill patients at risk for ARDS (controls). Compared with the controls, the ARDS patients had higher HNE, but lower PI3, at the onset of ARDS, resulting in increased HNE/PI3 ratio (mean = 14.5; 95% CI, 10.9–19.4, *P*<0.0001), whereas plasma SLPI was not associated with the risk of ARDS development. Although the controls had elevated plasma PI3 and HNE, their HNE/PI3 ratio (mean = 6.5; 95% CI, 4.9–8.8) was not significantly different from the healthy individuals (mean = 3.9; 95% CI, 2.7–5.9). Before the onset (7-days period prior to ARDS diagnosis), we only observed significantly elevated HNE, but the HNE-PI3 balance remained normal. With the progress from prior to the onset of ARDS, the plasma level of PI3 declined, whereas HNE was maintained at a higher level, tilting the balance toward more HNE in the circulation as characterized by an increased HNE/PI3 ratio. In contrast, three days after ICU admission, there was a significant drop of HNE/PI3 ratio in the at-risk controls.

**Conclusions/Significance:**

Plasma profiles of PI3, HNE, and HNE/PI3 may be useful clinical biomarkers in monitoring the development of ARDS.

## Introduction

Acute Respiratory Distress Syndrome (ARDS) is characterized by inflammation of the lung parenchyma leading to impaired gas exchange with concomitant systemic release of inflammatory mediators [1]. It is one of the most common disease processes in intensive care units (ICU), and is a major cause of morbidity and mortality in the ICU throughout the world [2]. Despite the development of clinical criteria for the diagnosis by American-European Consensus Conference (AECC) on ARDS, there remains a discrepancy between clinical criteria and histological findings with a substantial proportion of patients with clinical ARDS having no evidence of diffuse alveolar damage at autopsy [3]. Due to the lack of specificity of the clinical definition, there have been recent efforts to identify biological markers for diffuse alveolar damage in pulmonary edema fluid, blood and urine collected from critically ill patients with ARDS [4], [5], [6].

Previously we conducted an exploratory study of genome-wide gene expression in whole blood samples from ARDS patients using a repeated measures self-control study design to minimize noise [7]. By comparing expression between the acute-stage and recovery-stage of ARDS, we identified an ARDS-related gene encoding a potent human neutrophil elastase inhibitor (PI3, peptidase inhibitor 3, also known as elastase specific inhibitor, skin-derived antileukoprotease, or elafin), which was down-regulated during the acute-stage of ARDS. Analyses of plasma PI3 levels revealed a rapid decrease during early ARDS development, which was well correlated with PI3 gene expression. Neutrophil elastase (HNE) is one of the most destructive enzymes with the capability of degrading almost all extracellular matrix and key plasma proteins, and plays a crucial role in the pathophysiology of ARDS [8], [9]. We only observed HNE expression in few blood samples while majority of samples did not express this gene in the same study. This observation is consistent with previous findings that *HNE* gene is mainly expressed at the promyelocytic stage of granulocyte development, and mRNA cannot be detected in circulating neutrophils [10], [11].

A high concentration of HNE is stored in azurophil granules of neutrophils, providing a powerful host defense. Upon activation, however, HNE can be released rapidly into the extracellular space and cause tissue damage [9]. Endogenous proteinase inhibitors are important to protect tissues from unregulated proteolysis. Once released in circulation, HNE is rapidly inactivated by conjugating with protease inhibitors, mostly high-molecular-weight inhibitors including α1-antitrypsin and α2-macroglobulin [9]. Whereas low-molecular-weight inhibitors, such as elafin (PI3) and secretory leukocyte proteinase inhibitor (SLPI), are especially critical in local protection by their capability of penetrating into the “microenvironment” created by neutrophil to sequester HNE from high-molecular-weight inhibitors [12]. A local imbalance between proteinases and inhibitors results in pulmonary parenchyma damage by leakage of a protein-rich fluid into the interstitium and alveolar spaces, which is the major mechanism for activated neutrophils initiating and propagating ARDS [8].

PI3and SLPI are the only members of low-molecular-weight proteinase inhibitor family produced locally at the site of neutrophil infiltration such as lung [13], [14]. In contrast to SLPI, PI3 has a narrower spectrum of inhibition specifically toward HNE and proteinases 3 [14]. In addition, PI3 molecule has a unique N-terminal structure containing several transglutaminase substrate motifs that can bind the whole molecule to extracellular matrix (ECM) proteins via covalent cross-linking [15], and the covalently bound PI3 can still inhibit HNE [16]. Thus, PI3 can provide more efficient local protection against HNE in lung. It is very difficult to assess precisely the pulmonary level of PI3 in acute lung injury (ALI) or ARDS, as most of PI3 is anchored to lung parenchyma and exerts its biological functions locally [17]. We hypothesized that the plasma changes of low-molecular-weight proteinase inhibitors (PI3 and SLPI) and their antagonist HNE can be used as surrogate markers in monitoring the clinical progress of ARDS. More specifically, the disruption of the balance between proteinase inhibitors and HNE toward excessive HNE in circulation might be correlated with ARDS development. Therefore, we conducted a comprehensive investigation of plasma profiles of PI3, SLPI, and HNE in ARDS patients and at-risk, critically ill controls.

## Materials and Methods

### Objectives

We analyzed the plasma levels of PI3, SLPI, and HNE in ARDS patients and at-risk controls to investigate the hypothesis that plasma changes of proteinase inhibitors (PI3 and SLPI) and their antagonist HNE can be used as surrogate markers in monitoring the clinical progress of ARDS.

### Participants

This study was conducted within the ongoing project, the Molecular Epidemiology of ARDS, at the Massachusetts General Hospital (MGH) in Boston, Massachusetts, which is a prospectively-enrolled cohort study of ARDS [18]. The flowchart of study design is illustrated in Online Supplementary Material, Figure S1. Briefly, all patients enrolled from adult ICU at MGH are at risk for the development of ARDS with well-characterized predisposing clinical conditions, and are followed prospectively for the development of ARDS during their ICU stay [19], [20]. Predisposing clinical conditions are: 1) sepsis, 2) septic shock, 3) trauma, 4) pneumonia, 5) aspiration, or 6) massive transfusion of packed red blood cells (PRBC: defined as greater than 8 units of PRBC during the 24 hours prior to admission) as previously described [18]. Patients with previous history of ARDS and previous enrollment as controls were excluded. ARDS cases were defined by the AECC criteria [3]. Controls were identified as at-risk patients who did not meet criteria for ARDS during their ICU stay and had no prior history of ARDS. Exclusion criteria included age <18; diffuse alveolar hemorrhage; chronic lung diseases other than COPD or asthma; directive to withhold intubation; and treatment with immunosuppressive agent or immunoenhancing agent such as granulocyte colony-stimulating factor in the preceding 21 days.

### Ethics

The study was approved by the Human Subjects Committee of MGH. A written informed consent was obtained from each patient or an appropriate proxy.

### Clinical Data

Baseline clinical information, vital signs, and laboratory testing results in the first 24 hours of ICU admission were collected for calculation of the Acute Physiological and Chronic Health Evaluation (APACHE) III score [21]. In addition to a blood collection for DNA extraction during ICU stay, plasma samples were also collected for long-term storage.

### Plasma Sample Collection

Based on the original sampling protocol, plasma was collected from each enrolled patient within 48 hours of ICU admission, and a second sample was collected three days later. If an enrolled patient developed ARDS, two additional samples were collected corresponding to the first 48-hour of ARDS diagnosis and three days later. However, given the critical condition of ICU patients, difficulties in identifying surrogates and obtaining consent in time, the limitation of total blood drawn from each subject set by the IRB, as well as depletion from our previous study, only a fraction of enrolled patients had plasma available in this study (Online Supplementary Material, Figure S1). Since over 90% of ARDS cases developed ARDS during the first 7 days of ICU admission, the plasma from ARDS patients could be divided into three groups, the pre-ARDS samples (up to 7 days before ARDS diagnosis, n = 19), the ARDS samples (within 48 hours of diagnosis, n = 67), and the post-ARDS samples (day 2 to 4 of diagnosis, n = 105). In the current study, we excluded three samples collected more than 8 days before ARDS diagnosis and used the rest of available samples from ARDS cases, including 191 plasma samples from 148 ARDS patients. In addition, for the purpose of investigating temporal changes, we included paired plasma samples from the controls without ARDS, corresponding to the sampling time points within 48 hours of ICU admission and three days later after the first collection. Sixty-four control patients were selected randomly from 176 controls with paired plasma. Furthermore, 28 anonymous plasma samples collected from healthy male Caucasians were included as reference samples.

### ELISA Analysis of Plasma Profiles

Plasma samples were stored at −80°C until analysis. Plasma PI3, SLPI, and HNE levels were quantified in duplicate using Human pre-ELAFIN/SKALP (Cat. No. HK318), Human SLPI (Cat No. HK316), and Human Elastase (Cat No. HK319) ELISA Test Kit from Cell Sciences (Canton, MA), according to the manufacturer's recommended protocol. Except one control plasma sample which were excluded from the analyses, the CVs of replicates for the rest of samples were under 13%, which were below the manufacturer's recommended 15% standard. Eleven plasma samples were randomly selected as replicates for quality evaluation, and we had the inter-assay CVs ranging from 4% to 25%.

### Statistical analysis

The baseline characteristics between groups were compared using chi-square tests for categorical variables, and two sample *t*-tests for continuous variables. The normality of continuous variables was tested using Kolmogorov-Smirnov and Anderson-Darling methods. Since plasma profiles of PI3, SLPI, HNE, and HNE/PI3 ratio had skewed distributions, the natural log transformed data were applied to adjust normality in the analyses. Plasma profiles among different sample groups were compared by the two sample *t*-test, ANOVA (general linear model), and the mixed effect model for repeated samples. Paired *t*-test and Wilcoxon sign-rank test were used as well to analyze the paired samples from controls. In ANOVA analyses, Bonferroni correction was used to adjust for multiple comparisons. The relationship between ARDS plasma profiles and the sampling date relative to ARDS diagnosis was investigated by mixed effect models. Clinical relevant covariates were adjusted in the analyses, including age, gender, type of lung injury (pulmonary vs. extrapulmonary injury), pre-admission steroid use, septic shock, and APACHE III score on ICU admission. Patients with pneumonia, aspiration, pulmonary contusions, or sepsis from lower pulmonary source were categorized as pulmonary injury; whereas, patients with sepsis from an extrapulmonary source, trauma without pulmonary contusions, and multiple transfusions were categorized as extrapulmonary injury. Patients with both pulmonary and extrapulmonary injuries were considered to have pulmonary injury. All statistical analyses were performed by using the SAS statistical software package (version 9.1, SAS Inc., Cary, NC).

## Results

### Baseline Characteristics of the Study Population

Of 1,695 enrolled patients who had ARDS risk factors and no exclusion criteria by the end of 2007, 513 patients eventually developed ARDS during ICU hospitalization. Baseline characteristics between the selected ARDS cases and controls, as shown in Table 1, were similar to those published in previous studies within the similar study population [18], [22]. There were 191 plasma samples collected from 148 ARDS patients available in the present study. When the ARDS patients with plasma samples were compared to those without plasma samples, there were no significant differences with regard to age, gender, ethnicity, or other relevant baseline characteristics (*P*>0.05 for all comparisons). However, the studied ARDS cases had significantly higher proportions of pneumonia, pulmonary injury, and pre-admission steroid use, as well as higher APACHE III score on ICU admission (Online Supplementary Material, Table S1). Sixty-four critically ill patients with ARDS risk factors randomly selected as controls had baseline characteristics that were not significantly different from the unselected controls (*P*>0.05 for all comparisons) except that selected controls had a higher APACHE III score on ICU admission (*P* = 0.027) (Online Supplementary Material, Table S2). Since the paired plasma from one control failed to generate reliable plasma profiles, we excluded this subject resulting in 63 controls in the analyses. Removing this control did not result in significant changes in the baseline characteristics of the controls (data not shown). The reference group included anonymous plasma samples from male healthy donors, which were significant younger than the studied population (mean age±SD: 40±11; *P*<0.0001).

**Table 1:** Characteristics of the study population

	ARDS (n = 148)	At-risk controls (n = 64)	*P*
**Age-yr, mean±SD**	60±19	61±17	0.730
**Gender, male/female**	90/58	38/26	0.844
**Caucasian, n (%)**	140 (94.4)	61 (95.3)	0.829
**APACHE III score, mean±SD** a	81±22	72±23	**0.006**
**On ventilation at ICU admission, n (%)**	131 (88.5)	40 (62.5)	<**0.0001**
**Risk factors, n (%)**			
Sepsis	134 (90.5)	52 (81.3)	0.058
Septic shock	89 (60.1)	28 (43.8)	**0.028**
Pneumonia	118 (79.7)	25 (39.1)	<**0.0001**
Aspiration	14 (9.5)	6 (9.4)	0.985
Pulmonary injury^b^	125 (84.5)	31 (48.4)	<**0.0001**
Multiple transfusion	10 (6.8)	7 (10.9)	0.304
Trauma	7 (4.7)	6 (9.4)	0.196
**Comorbidities, n (%)**			
Diabetes	28 (18.9)	15 (23.4)	0.452
Liver failure/cirrhosis	10 (6.8)	3 (4.7)	0.564
**Corticosteroid treatment before ICU admission, n (%)** c	26 (17.6)	4 (6.3)	**0.030**

ARDS, acute respiratory distress syndrome; APACHE, Acute Physiology and Chronic Health Evaluation;

a. APACHE III physiology score was calculated with all components on the day of ICU admission;

b. Pneumonia, aspiration, pulmonary contusions, or sepsis from lower pulmonary source were categorized as pulmonary injury. Sepsis from an extrapulmonary source, trauma without pulmonary contusions, and multiple transfusions were categorized as extrapulmonary injury. Patients with both pulmonary and extrapulmonary injuries were considered to have pulmonary injury;

c. Patient received ≥ 300 mg of prednisone or its equivalent within 21 days or ≥ 15 mg prednisone a day or its equivalent prior to ICU admission.

### Correlations among Plasma Profiles in the ARDS and the Control

Plasma profiles of PI3, SLPI, and HNE on 67 ARDS plasma samples collected within 48-hour of diagnosis were compared with the profiles on 63 control samples collected within 48-hour of ICU admission. The correlation matrix of plasma profiles and APACHE III scores were shown in Online Supplementary Material, Table S3. Plasma PI3 correlated with plasma SLPI in the ARDS samples (Pearson correlation coefficient: ρ = 0.37, *P* = 0.004) and in the control samples where the correlation was stronger (ρ = 0.50, *P*<0.0001). In contrast, plasma PI3 had a moderate correlation with plasma HNE in the ARDS sample (ρ = 0.42, *P* = 0.0009), but not in the control sample (ρ = 0.03, *P* = 0.815) (Online Supplementary Material, Figure S2). In both the ARDS and the control, plasma PI3 and SLPI demonstrated moderate correlations with APACHE III score on ICU admission (ρ = 0.34–0.51, *P*<0.01 for all analyses), but plasma HNE only showed correlation with APACHE III score in the ARDS samples (ρ = 0.51, *P*<0.0001). Since plasma PI3 and HNE showed some different correlations between the ARDS samples and the control samples, we proposed to combine two variables by calculating the ratio of HNE/PI3 and included it in the subsequent analyses. The HNE/PI3 ratio was not correlated with APACHE III score in both ARDS cases and controls.

### Comparison of Plasma Profiles between ARDS and Controls

As shown in Table 2, both the ARDS samples (n = 67, 48-hour of ARDS diagnosis) and the ICU control samples (n = 63, 48-hour of ICU admission) had significantly elevated plasma levels of PI3, SLPI, and HNE (*P*<10^−6^ for all comparisons, two sample *t*-tests), as compared with the reference plasma samples from healthy individuals (n = 28). When compared with the controls on ICU admission in two sample *t*-tests, there were significantly higher levels of plasma HNE (*P* = 0.007) and HNE/PI3 ratio (*P* = 0.001) at ARDS diagnosis, but no significant difference in plasma SLPI (*P* = 0.657). The ARDS samples had a lower plasma PI3 but did not show statistical significance (*P* = 0.115). After adjusting for age, gender, type of lung injury, septic shock, pre-admission steroid use, and APACHE III score on ICU admission, there was a significantly lower level of plasma PI3 in the ARDS, whereas the remaining test results were unchanged (Table 2). The opposite changes of PI3 and HNE between the ARDS and the control, which resulted in a highly increased HNE/PI3 ratio, suggested that the balance between neutrophil elastase and its inhibitor was severely damaged with elastase dominant in blood at the onset of ARDS. In contrast, the HNE/PI3 ratio was not statistically significant different between the controls and the reference samples (two sample *t*-test, *P* = 0.105).

**Table 2:** Plasma profile comparison between ARDS and controls

	ARDS Mean (95% CI)	Control Mean (95%CI)	*P* c	Healthy Reference Mean (95%CI) d
**All samples** a	N = 67	N = 63		
PI3	44.3 (35.0–56.0)	69.3 (54.4–88.2)	**0.005**	19.6 (13.9–27.5)
SLPI	115.3 (101.2–131.5)	132.5 (115.8–151.7)	0.752	69.6 (62.3–77.9)
HNE	643.6 (525.3–788.7)	453.3 (367.6–558.9)	**0.017**	77.1 (63.9–93.1)
HNE/PI3 ratio	14.5 (10.9–19.4)	6.5 (4.9–8.8)	**<0.0001**	3.9 (2.7–5.9)
**Pulmonary injury** b	N = 54	N = 30		
PI3	44.6 (35.6–55.9)	81.7 (60.2–110.8)	**0.001**	
SLPI	126.2 (112.0–142.3)	140.7 (119.6–165.4)	0.221	
HNE	546.2 (447.5–666.7)	396.3 (302.6–519.1)	0.107	
HNE/PI3 ratio	12.2 (9.3–16.1)	4.9 (3.3–7.0)	**<0.0001**	
**Extrapulmonary injury** b	N = 13	N = 33		
PI3	66.7 (36.2–122.9)	77.9 (44.9–135.3)	0.480	
SLPI	108.6 (74.0–159.3)	136.9 (96.9–193.5)	0.655	
HNE	639.9 (376.7–1087.1)	491.0 (304.5–791.6)	0.655	
HNE/PI3 ratio	13.7 (4.5–20.3)	6.3 (3.2–12.4)	0.254	

ARDS, acute respiratory distress syndrome; Control, at-risk ICU control; PI3, neutrophil elastase inhibitor (elafin); SLPI, secretory leukocyte proteinase inhibitor; HNE, neutrophil elastase; the plasma levels of PI3, SLPI, and HNE were shown in unit of ng/ml; 95%CI, 95% confidence interval.

a. To account for repeated measurements, mean plasma levels were calculated using mixed models after adjusting for age, gender, type of lung injury, pre-admission steroid use, septic shock, and APACHE III score on ICU admission. Type of lung injury: pulmonary injury - pneumonia, aspiration, pulmonary contusions, or sepsis from lower pulmonary source; extrapulmonary injury - sepsis from an extrapulmonary source, trauma without pulmonary contusions, and multiple transfusions. Patients with both pulmonary and extrapulmonary injuries were considered to have pulmonary injury.

b. To account for repeated measurements, mean plasma levels were calculated using mixed models after adjusting for age, gender, pre-admission steroid use, septic shock and APACHE III score on ICU admission.

c. *P* values were calculated between the ARDS and the controls.

d. Mean plasma levels of anonymous healthy individuals (reference groups) were calculated directly without any adjustment.

In subgroup analyses within different types of lung injury, we only found similar significant changes in plasma profiles of PI3 and HNE in the pulmonary injury group, but not in the extrapulmonary injury group (Table 2). Moreover, either in the ARDS or in the controls, we did not observe any significant difference of plasma profiles between different types of lung injury.

### Plasma profiles among pre-ARDS, ARDS and controls

**Figure 1:**
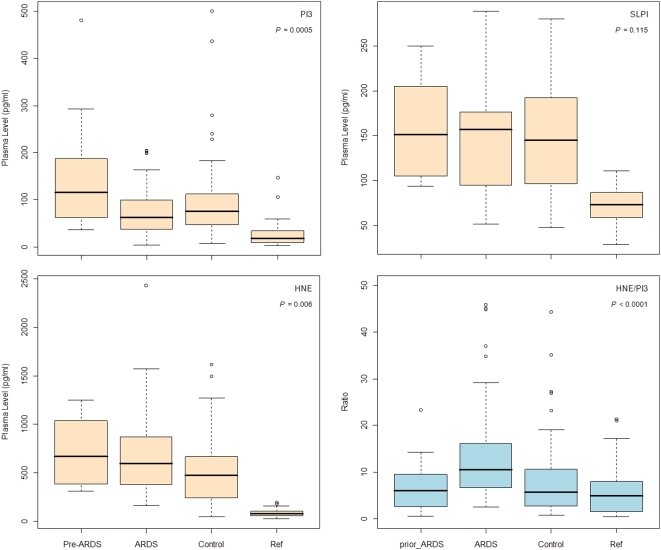
Box plot of plasma profiles of PI3, SLPI, HNE, and HNE/PI3 among pre-ARDS, the ARDS and the controls. Pre-ARDS: plasma sample (n = 19) collected during 7-day period before ARDS diagnosis; ARDS: samples (n = 67) collected within 48-hour of ARDS diagnosis; Control: samples (n = 63) collected within 48-hour of ICU admission; Ref: anonymous plasma samples from healthy individual (n = 28). The plasma profiles among three groups were tested by general linear model with adjustment of age, gender, pulmonary injury, pre-admission steroid use, and higher APACHE III score on ICU admission (*P* values showed in the plots).

To explore the usefulness of using plasma profiles to predict the risk of ARDS development, we focused initially on the samples collected on ICU admission (first 48-hour). Only a fraction of ARDS patients were diagnosed during the first 48-hour of ICU admission, whereas, the rest were diagnosed later during the ICU hospitalization. Thus, samples collected on ICU admission could be classified into three groups, including pre-ARDS, ARDS and control patients who did not develop ARDS during ICU stay. However, out of 191 ARDS samples available in this study, we only identified 28 plasma samples which had been collected on ICU admission, including 7 pre-ARDS samples and 21 ARDS samples. Considering relatively small number of samples in the pre-ARDS group and ARDS group collected on ICU admission, we combined all available pre-ARDS samples (n = 19) and ARDS (within 48-hour diagnosis, n = 67) in the analyses. The plasma PI3, HNE and HNE/PI3 were statistically significant different among three groups in both crude analyses (ANOVA, *P* = 0.004, 0.007, and 0.0004, respectively), and in the adjusted analyses with age, gender, pulmonary injury, pre-admission steroid use, and higher APACHE III score on ICU admission (general linear model, *P* = 0.0005, 0.006, and <0.0001, respectively). There was no significant difference of plasma SLPI among three groups in crude analysis (ANOVA: *P* = 0.943) and adjusted analysis (general linear model, *P* = 0.115). In pairwise comparisons with Bonferroni correction for multiple testing, firstly, we found similar changes of plasma profiles between the ARDS and the controls, with plasma HNE and HNE/PI3 ratio significant higher in the ARDS, but PI3 did not reach significance after correction for multiple comparisons (Figure 1). Secondly, when comparing the pre-ARDS and the ARDS, the ARDS group had significantly lower plasma PI3 but a higher HNE/PI3 ratio than the pre-ARDS group. However, there was no significant difference in HNE between the pre-ARDS and the ARDS samples. Finally, the plasma HNE in pre-ARDS was significantly higher than the controls (*P* = 0.018), but no significant difference of plasma PI3, SLPI, and HNE/PI3 ratio was observed between two groups (Table 3). Furthermore, although the pre-ARDS had significantly higher PI3, SLP, and HNE than the reference samples (*P*<10^−6^ for all comparisons, two sample *t*-tests), the HNE/PI3 ratio was not significantly different between two groups (*P* = 0.276, two sample *t*-tests). In summary, these findings indicated that the balance between PI3 and HNE was maintained before the onset of ARDS.

**Table 3:** ANOVA analyses of plasma profiles among the pre-ARDS, ARDS and the Control^a^

	Control	Pre-ARDS	ARDS	Mean (95% CI) ng/ml^c^	*P* b
**PI3**					**0.009**
Control	NA	++	+	80.3 (66.7–96.6)	
Pre-ARDS		NA	++	87.5 (61.0–125.5)	
ARDS			NA	52.9 (42.7–65.6)	
**SLPI**					0.187
Control	NA	-	-	139.4 (126.1–154.0)	
Pre-ARDS		NA	-	125.2 (103.6–151.4)	
ARDS			NA	120.7 (107.6–135.5)	
**HNE**					**0.012**
Control	NA	+	++	447.5 (380.6–526.2)	
Pre-ARDS		NA	-	643.4 (472.8–875.7)	
ARDS			NA	633.0 (524.8–763.6)	
**HNE/PI3**					**0.0001**
Control	NA	-	++	5.6 (4.4–7.0)	
Pre-ARDS		NA	++	7.5 (4.8–11.7)	
ARDS			NA	12.0 (9.2–15.6)	

ARDS, acute respiratory distress syndrome; PI3, neutrophil elastase inhibitor (elafin); SLPI, secretory leukocyte proteinase inhibitor; HNE, neutrophil elastase; 95%CI, 95% confidence interval.

a. ANOVA analysis plasma profiles using general linear model with adjustment of covariates, including age, gender, type of lung injury, pre-admission steroid use, septic shock, and APACHE III score on ICU admission. Pairwise comparison: ++, P<0.05 after Bonferroni correction for multiple comparisons; +, *P*<0.05 without Bonferroni correction; -, *P*≥0.05; NA, not applicable.

b. *P* values of general linear model for all groups.

c. The unit of HNE/PI3 is fold change.

### Decrease of plasma PI3 with the clinical progress of ARDS

**Figure 2:**
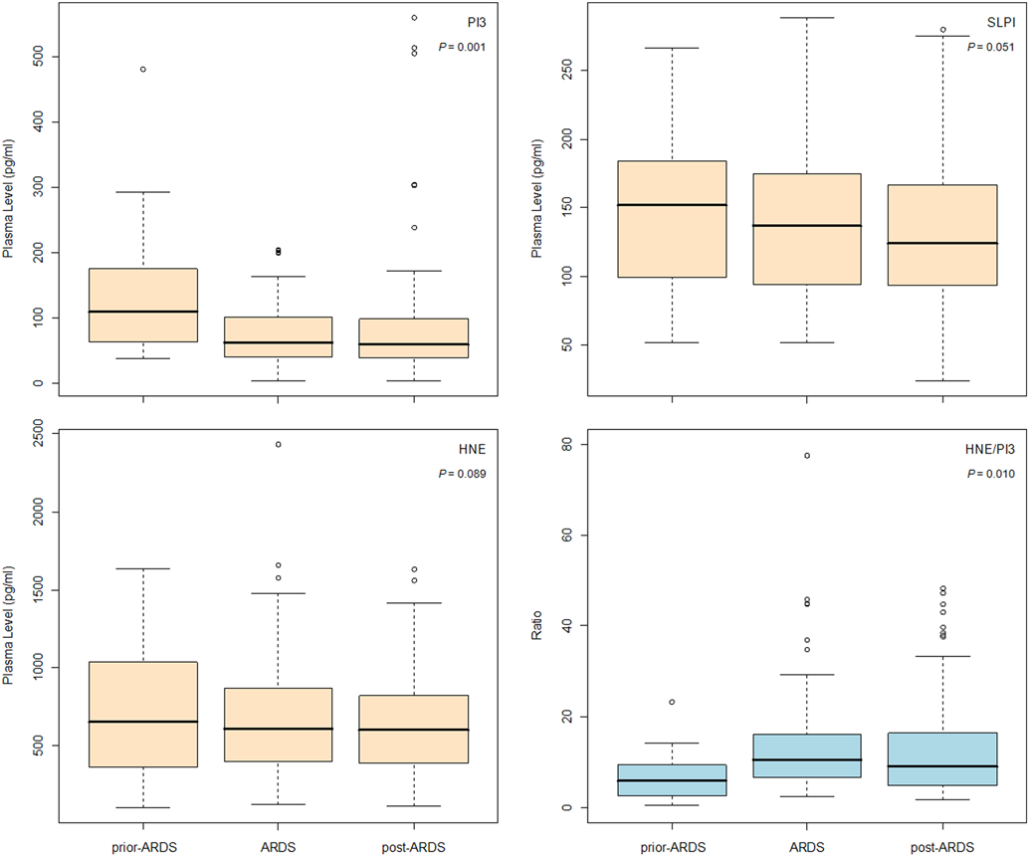
Box plot of plasma profile changes in clinical progress of ARDS. Pre-ARDS: plasma sample (n = 19) collected during 7-day period before ARDS diagnosis; ARDS: samples (n = 67) collected within 48-hour of ARDS diagnosis; Post-ARDS: samples (n = 105) collected between Day 2 and Day 4 of ARDS diagnosis. The trends of plasma profiles across ARDS development, as measured by reciprocal of days between sampling dates relative to ARDS diagnosis, were tested by mixed effect models, adjusted for age, gender, type of pulmonary injury, pre-admission steroid use, and APACHE III score on ICU admission (*P* values showed in the plots).

Our previous results suggested that reduction of PI3 along with the clinical progress of ARDS might be the cause for disrupting the HNE-PI3 balance. Next, we looked the changes of plasma profiles among three sample groups from ARDS patients, including pre-ARDS, at ARDS diagnosis, and post-ARDS diagnosis. The PI3 and HNE/PI3 were significant changed among three groups in both the crude analyses (ANOVA, *P* = 0.004 and *P* = 0.010, respectively), and in the analyses adjusted for age, gender, pulmonary injury, pre-admission steroid use, and APACHE III score on ICU admission (general linear model, *P* = 0.015 and *P* = 0.036, respectively). The pre-ARDS samples had significant higher PI3, but lower HNE/PI3, than either the ARDS or the post-ARDS in pairwise comparisons after correction for multiple testing (Figure 2). In contrast, there was no difference in plasma SLPI and HNE among three samples groups. We further tested the trend of plasma PI3 decrease across ARDS development, as measured by sampling dates relative to ARDS diagnosis, using mixed effect models. The reciprocal of days between sampling dates relative to ARDS diagnosis was significantly related to plasma PI3 and HNE/PI3 (mixed models, *P* = 0.001 and *P* = 0.010, respectively), suggesting that the closer to ARDS onset, the greater the decrease in PI3 and the higher degree of HNE/PI3 imbalance.

### Changes of plasma profiles between paired samples of controls

The changes in plasma profiles in control patients who did not develop ARDS during ICU stay were investigated between paired samples collected within 48 hours of ICU admission and three days after the first sample collection (Table 4). As compared with the ICU admission, the second plasma had significant lower HNE (paired *t*-test, *P* = 0.003; Wilcoxon sign-rank test, *P* = 0.0008), accompanied by a lower HNE/PI3 ratio (paired *t*-test, *P* = 0.001; Wilcoxon sign-rank test, *P* = 0.0004). This observation suggests that the elevated plasma HNE was decreased in those patients who did not develop ARDS. Interestingly, we found the plasma profiles changed differently by type of lung injury, while the final results were in favor of maintaining enough inhibition capacity against HNE. In the patients with pulmonary injury, there was a significant decrease of HNE (*P* = 0.023) without significant changes in PI3 (*P* = 0.302). Conversely in the patients with extrapulmonary injury, there was a significant increase of PI3 (*P* = 0.023) without significant changes in HNE (*P* = 0.069).

**Table 4:** Changes of plasma profiles between paired samples

	First Sample a Mean (95% CI)	Second Sample b Mean (95%CI)	*P* c
**Control (all samples, n = 63)**			
PI3	73.1 (58.9–90.7)	76.9 (63.4–93.3)	0.335
SLPI	133.6 (118.8–150.1)	119.7 (106.8–134.2)	**0.019**
HNE	421.8 (351.5–506.1)	329.8 (276–394.1)	**0.003**
HNE/PI3 ratio	5.8 (4.4–7.6)	4.3 (3.3–5.5)	**0.001**
**Control (pulmonary injury, n = 30)**			
PI3	88.2 (62.3–124.7)	81.6 (58.4–113.9)	0.302
SLPI	147 (125.4–172.2)	133.9 (115.4–155.4)	0.164
HNE	351.8 (268.6–460.7)	262.7 (202.5–340.9)	**0.023**
HNE/PI3 ratio	4.0 (2.6–6.2)	3.2 (2.1–4.9)	0.115
**Control (extrapulmonary injury, n = 33)**			
PI3	61.6 (47–80.7)	73 (58.1–91.6)	**0.023**
SLPI	122.4 (103–145.5)	108.1 (91.1–128.3)	0.063
HNE	497.4 (388.8–636.5)	405.6 (321.1–512.4)	0.069
HNE/PI3 ratio	8.1 (5.8–11.3)	5.6 (4.1–7.5)	**0.004**
**ARDS (all samples, n = 24)**			
PI3	48.9 (31.4–76)	57.5 (38.9–85.1)	0.132
SLPI	122.1 (101.3–147.1)	130.5 (104–163.9)	0.226
HNE	561 (414.4–759.4)	522.5 (421.3–648)	0.552
HNE/PI3 ratio	11.5 (7.7–17)	9.1 (6.1–13.6)	0.083

PI3, neutrophil elastase inhibitor (elafin); SLPI, secretory leukocyte proteinase inhibitor; HNE, neutrophil elastase; the plasma levels of PI3, SLPI, and HNE were shown in unit of ng/ml; 95%CI, 95% confidence interval.

a. For at-risk control patients, the first plasma samples were collected within 48-hour of ICU admission; For ARDS patients, the first plasma samples were collected within 48-hour of ARDS diagnosis.

b. The second samples were collected three days after the first collection.

c. Paired *t*-test

Twenty-four ARDS patients provided paired plasma in the study, with the first sample collected during the first 48 hours of ARDS diagnosis and second sample collected three days later. However, there was no significant change in PI3 (*P* = 0.132), SLPI (*P* = 0.226), HNE (*P* = 0.552), and HNE/PI3 (*P* = 0.083) between two time points.

## Discussion

Previous research showed that a local imbalance between proteinases and their physiological inhibitors damage the pulmonary parenchyma resulting in leaking a protein-rich fluid into the interstitium and alveolar spaces, a process that plays a crucial role in the initiation and propagation of ARDS [9]. Our study extends this observation into the peripheral circulation. At the onset of ARDS, the ARDS patients had significantly higher HNE but lower PI3 resulting in increased HNE/PI3 ratio (mean = 14.5; 95% CI, 10.9–19.4), compared with patients who did not develop ARDS during the ICU hospitalization which had a higher but not statistically significant HNE/PI3 ratio (mean = 6.5; 95% CI, 4.9–8.8) than that of healthy individuals (reference samples: mean = 3.9; 95% CI, 2.7–5.9). Before the ARDS onset (7-day period prior to ARDS diagnosis), we observed only a significantly elevated HNE, but an unchanged HNE/PI3 ratio as compared with either the at-risk controls at the ICU admission or the healthy reference group. With the progress of acute lung injury from prior to the onset of ARDS, the PI3 plasma level was dropped whereas HNE was maintained at a higher level, tilting the balance toward more neutrophil elastase in the circulation characterized by increased HNE/PI3 ratio. In contrast, three days after ICU admission, there was a significant drop of the HNE/PI3 ratio in the at-risk controls. Therefore, these results suggest that the loss of the balance between plasma neutrophil elastase (HNE) and its specific inhibitor elafin (PI3) is related to the risk of ARDS development. Furthermore, the plasma profiles of PI3, HNE, and HNE/PI3 can be used as clinical biomarkers in monitoring the development of ARDS. However, plasma SLPI was not associated with the risk of ARDS development.

Although both PI3 and SLPI are low-molecular-weight proteinase inhibitors belonging to the same chelonianin family of canonical inhibitors (Family I17 Clan IP in the MEROPS database), PI3 has a much narrower anti-proteinase spectrum since it inhibits only neutrophil elastase and proteinase 3 [14]. In addition, PI3 has a unique N-terminal non-inhibitory domain, containing several transglutaminase substrate motifs, which can covalently anchor the whole molecule to extracellular matrix (ECM) protein through protein cross-linking [15], [16]. Small molecular size, specific anti-elastase spectrum, and immobilization to ECM are major characteristics that determine how PI3 plays critical protective roles against the tissue damage induced by HNE during acute lung injury. The expression of *PI3* gene can be readily induced under inflammatory conditions by pro-inflammatory cytokines, such as IL-β1 and TNF-α, and elastase [23], [24], [25]. Our previous genome-wide gene expression analysis revealed that *PI3* gene demonstrated the largest down-regulation in peripheral blood expression at the early stage of ARDS, as compared with the recovery stage around ICU discharge [7]. In a smaller set of samples (40 ARDS patients and 23 at-risk controls), the protein expression in plasma correlated well with the microarray findings, with a lower level of plasma PI3 during the acute-stage. In the same study, we were first to report that there was a trend of plasma PI3 decreasing from pre- to post-diagnosis of ARDS. The results of this study confirmed our previous study by observing the same PI3 decreasing trend along with clinical progress toward ARDS development, with a larger sample size within the same study population (148 ARDS patients and 63 controls). We conducted additional analyses of plasma PI3 by pooling data from both studies, which had overlapped samples collected from 24 ARDS patients and 13 at-risk controls, and obtained consistent results (data not shown).

At another end of proteinase-inhibitor balance is the HNE, which has been implicated in the pathogenesis of ALI/ARDS in previous studies [26]. Elevated HNE was observed in animal models of ALI. Administration of HNE could induce typical symptoms of ALI in experiment animals, and inhibition of increased HNE could reduce ALI symptoms [9], [27]. Despite earlier clinical studies revealing conflicted results of elevated HNE in bronchoalveolar lavage fluid [8], [28], [29], more recent studies measuring plasma HNE found consistently increased plasma HNE in ALI/ARDS patients [30], [31], [32], [33]. Our observations of significantly higher plasma HNE in the ARDS patients were consistent with the previous research. In one particular study examining sequential blood samples, Kodama *et al*. reported that the plasma HNE level of ALI/ARDS patients (n = 18) with inflammatory response syndrome (SIRS) was significantly greater than that of SIRS alone (n = 5) [34]. A cut off value of >220 ng/ml was also proposed in the same study, as HNE in all patients with SIRS alone was consistently less than this value and all SIRS patients with HNE more than 220 ng/ml eventually developed ALI/ARDS. Because of a small sample size, that study pooled ALI and ARDS together as the case group to compare with a patient group having less severe conditions. This may explain why the majority of controls in our study had plasma HNE higher than the Kodama's cut off value, as many control patients in our study already developed ALI. Nevertheless, our findings that significant elevated HNE before the onset of ARDS (the pre-ARDS) agrees with a previous report that the onset of pulmonary dysfunction followed HNE increase.

Although previous studies suggest that the circulating HNE could be used as a predictive factor for ALI/ARDS development, patients with predisposing risk conditions for ARDS often had increased plasma HNE with a large range of variation. Similarly, despite the fact that we observed a significant lower level of plasma PI3 in the ARDS patients, there was a wide range of variation in both ARDS patients and the at-risk controls. PI3 and HNE are located at the opposite ends of the proteinase-inhibitor balance, and their expression levels could be affected by common as well as independent factors. In clinical practice, it is difficult to define a clear cutoff point to distinguish ARDS patients from those critically ill patients who did not develop ARDS by monitoring a single biomarker, which only depicts a half picture of the balance. Instead, simultaneous measurement of a group of functionally related biomarkers will be more practical to assess comprehensive status of ARDS. Since it is impractical to assess local HNE-PI3 balance in lung, monitoring the plasma HNE-PI3 balance is a good alternative.

We propose that the biological mechanism for our results is that, with the progress of pulmonary inflammation, the increased permeability allows free passing of HNE and PI3 through pulmonary blood-gas barrier. The change of plasma PI3 could be explained by the redistribution of PI3 from blood stream to local site. It is more likely that PI3 has a net unidirectional flow from blood to lung parenchyma as PI3 will be trapped in ECM by transglutaminase-catalyzed cross-links. Elevated circulating PI3 acts as an extra-pulmonary resource for protecting unregulated proteolysis. When plasma PI3 drops to certain level, HNE-PI3 balance cannot be maintained in both circulation and lung with the occurrence of ARDS. Additional studies are needed to test this hypothesis. Furthermore, this hypothesis may explain why a large multicenter clinical trial of sivelestat, a specific elastase inhibitor, failed to improve the survival rate of ALI/ARDS [35]. In that study, all patients were enrolled after the onset of ALI, which could have been too late for the action of an elastase inhibitor. In another smaller clinical trial, sivelestat was administrated from the ICU admission resulting in significant reduction of mortality in critically ill patients [36]. On the other hand, our hypothesis also suggests that PI3 itself may be a drug candidate for specific ARDS treatment.

All patients enrolled in the present study were at risk for the development of ARDS because of well-characterized predisposing clinical conditions, and were followed prospectively for the development of ARDS during their ICU hospitalization. This unique feature of the study design allowed us to investigate plasma profiles before the onset of ARDS. However, we acknowledge a limitation that the sample size of this study was relative small, especially in the pre-ARDS group. In addition, although paired sampling is a powerful study design in controlling the confounders, most of the repeated samples from the ARDS patients were the ARDS samples and the post-ARDS samples, without many the pre-ARDS sample. Thus, confirmatory studies with large sample sizes are required to validate this study. Moreover, we only measured the amount of total HNE in circulation, which was mostly conjugated with high-molecular-weight inhibitors, instead of assessing directly the HNE activity. Measuring HNE-inhibitor complex is commonly used for monitoring circulating HNE activity, as there is a high correlation between them [37]. Nevertheless, the level of conjugated complex between HNE and high-molecular-weight inhibitors did not systematically correlate with severity of illness [8].

## Supporting Information

Figure S1 Flow diagram of study design and sample selection. All admissions to the adult ICUs at the MGH in Boston, Massachusetts were screened on a daily basis to identify and recruit patients with one or more clinical conditions (risk factors) that are associated with ARDS, including sepsis, septic shock, pneumonia, trauma (multiple fractures and/or pulmonary contusions), multiple transfusions, and aspiration. All recruited patients were then followed on a daily basis for the development of ARDS, according to the AECC definition. All chest x-rays had been interpreted by two physicians blinded to the clinical conditions of the patient and any disagreement were decided by a third physician. Cases were identified as those who developed ARDS at anytime during their ICU stay, and controls were those who did not develop ARDS during their ICU hospitalization with no previous history of ARDS. According to sampling protocol, plasma was collected from each enrolled patient within 48 hours of ICU admission, and a second sample was collected three days later. If an enrolled patient developed ARDS, two additional samples were collected corresponding to the first 48-hour of ARDS diagnosis and three days later. However, given the critical condition of ICU patients, difficulties in identifying surrogates and obtaining consent in time, the limitation of total blood drawn from each subject set by the IRB, and the use of plasma samples in previous studies, only fraction of enrolled subjects had plasma samples available for this study.
(0.08 MB TIF)

Figure S2 Plot of plasma PI3 against HNE. Plasma PI3 and HNE were log transformed. ARDS: 67 samples collected within 48-hour of ARDS diagnosis; Control: 63 samples collected within 48-hour of ICU admission; Ref: 28 anonymous plasma samples from healthy individual.
(0.06 MB TIF)

Table S1 Compare characteristics between ARDS cases with plasma samples and those without plasma (excluded in this study)
(0.06 MB DOC)

Table S2 Compare characteristics between at-risk controls selected in this study and those excluded
(0.06 MB DOC)

Table S3 Correlation coefficients (p value) among plasma profiles and APACHE III score
(0.07 MB DOC)
