# Supplementary figures and images for: Plasma Neutrophil Elastase and Elafin Imbalance Is Associated with Acute Respiratory Distress Syndrome (ARDS) Development
Source: PLoS One. 2009 Feb 6;4(2):e4380. doi: 10.1371/journal.pone.0004380 (PMC2633615; doi:10.1371/journal.pone.0004380)

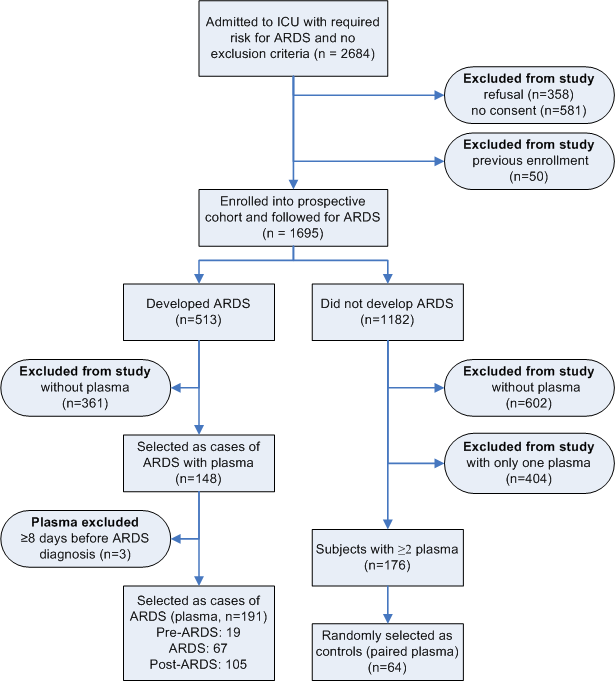

Supplement: Figure S1 — Flow diagram of study design and sample selection. All admissions to the adult ICUs at the MGH in Boston, Massachusetts were screened on a daily basis to identify and recruit patients with one or more clinical conditions (risk factors) that are associated with ARDS, including sepsis, septic shock, pneumonia, trauma (multiple fractures and/or pulmonary contusions), multiple transfusions, and aspiration. All recruited patients were then followed on a daily basis for the development of ARDS, according to the AECC definition. All chest x-rays had been interpreted by two physicians blinded to the clinical conditions of the patient and any disagreement were decided by a third physician. Cases were identified as those who developed ARDS at anytime during their ICU stay, and controls were those who did not develop ARDS during their ICU hospitalization with no previous history of ARDS. According to sampling protocol, plasma was collected from each enrolled patient within 48 hours of ICU admission, and a second sample was collected three days later. If an enrolled patient developed ARDS, two additional samples were collected corresponding to the first 48-hour of ARDS diagnosis and three days later. However, given the critical condition of ICU patients, difficulties in identifying surrogates and obtaining consent in time, the limitation of total blood drawn from each subject set by the IRB, and the use of plasma samples in previous studies, only fraction of enrolled subjects had plasma samples available for this study. (0.08 MB TIF) [file pone.0004380.s001.tif]

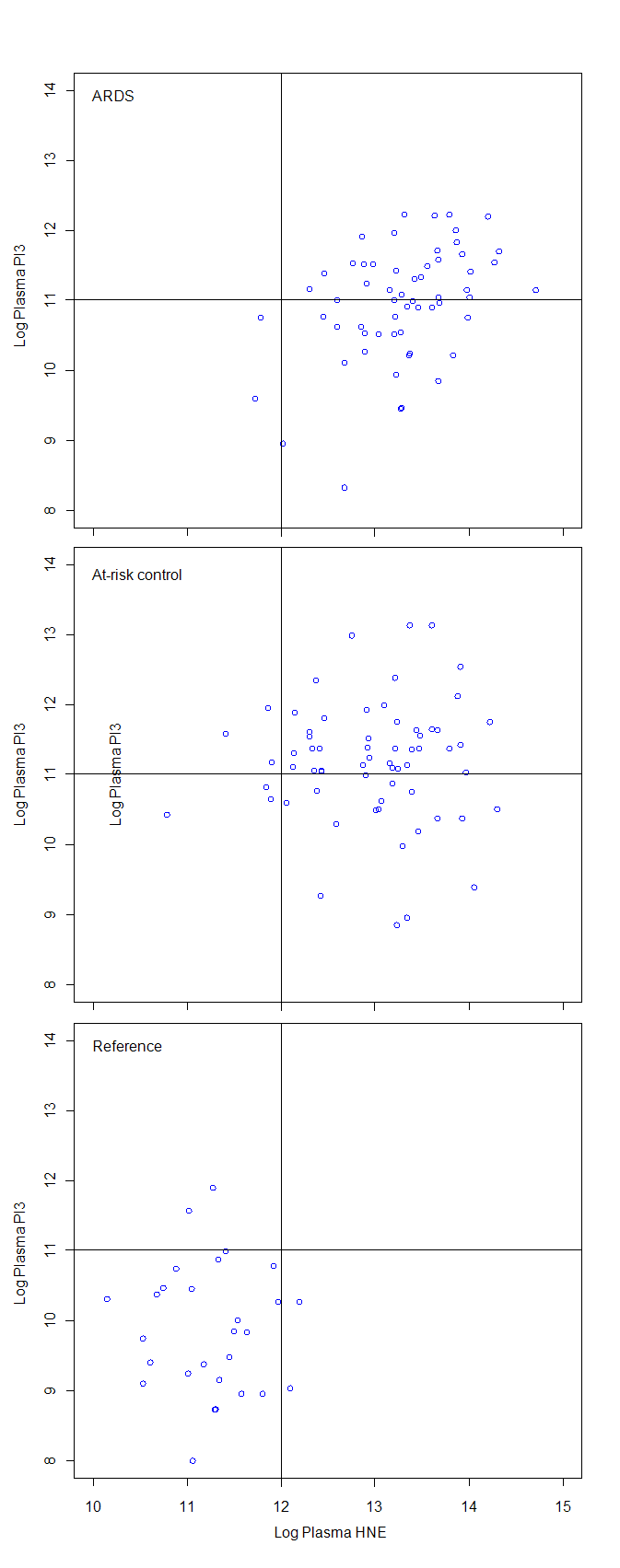

Supplement: Figure S2 — Plot of plasma PI3 against HNE. Plasma PI3 and HNE were log transformed. ARDS: 67 samples collected within 48-hour of ARDS diagnosis; Control: 63 samples collected within 48-hour of ICU admission; Ref: 28 anonymous plasma samples from healthy individual. (0.06 MB TIF) [file pone.0004380.s002.tif]
