# Supplementary material for: Plasma Neutrophil Elastase and Elafin Imbalance Is Associated with Acute Respiratory Distress Syndrome (ARDS) Development
Source: PLoS One. 2009 Feb 6;4(2):e4380. doi: 10.1371/journal.pone.0004380 (PMC2633615; doi:10.1371/journal.pone.0004380)
Supplement: Table S1 — Compare characteristics between ARDS cases with plasma samples and those without plasma (excluded in this study) (0.06 MB DOC) [file pone.0004380.s003.doc]

**Table S1. Compare characteristics between ARDS cases with plasma samples and those without plasma (excluded in this study)**

|  | **With Plasma  (n = 148)** | **Without Plasma  (n = 365)** | ***P*** |
| --- | --- | --- | --- |
| **Age-yr, mean ± SD** | 60 ± 19 | 59 ± 18 | 0.459 |
| **Gender, male/female** | 90/58 | 217/148 | 0.776 |
| **Caucasian, n (%)** | 140 (94.4) | 329 (90.1) | 0.102 |
| **APACHE III score, mean ± SD*^a^*** | 81 ± 22 | 76 ± 24 | **0.021** |
| **On ventilation at ICU admission, n (%)** | 131 (88.5) | 316 (86.6) | 0.553 |
| **Risk factors, n (%)** |  |  |  |
| Sepsis | 134 (90.5) | 309 (84.7) | 0.079 |
| Septic shock | 89 (60.1) | 210 (57.5) | 0.588 |
| Pneumonia | 118 (79.7) | 244 (66.9) | **0.004** |
| Aspiration | 14 (9.5) | 41 (11.2) | 0.556 |
| Pulmonary injury*^b^* | 125 (84.5) | 263 (72.1) | **0.003** |
| Multiple transfusion | 10 (6.8) | 41 (11.2) | 0.125 |
| Trauma | 7 (4.7) | 31 (8.5) | 0.140 |
| **Comorbidities, n (%)** |  |  |  |
| Diabetes | 28 (18.9) | 75 (20.7) | 0.656 |
| Liver failure/cirrhosis | 10 (6.8) | 27 (7.4) | 0.799 |
| **Corticosteroid treatment before ICU admission, n (%)*^c^*** | 26 (17.6) | 28 (7.7) | **0.001** |

ARDS, acute respiratory distress syndrome; APACHE, Acute Physiology and Chronic Health Evaluation;

1. APACHE III physiology score was calculated with all components on the day of ICU admission;
2. Pneumonia, aspiration, pulmonary contusions, or sepsis from lower pulmonary source were categorized as pulmonary injury. Sepsis from an extrapulmonary source, trauma without pulmonary contusions, and multiple transfusions were categorized as extrapulmonary injury. Patients with both pulmonary and extrapulmonary injuries were considered to have pulmonary injury;
3. Patient received ≥ 300 mg of prednisone or its equivalent within 21 days or ≥ 15 mg prednisone a day or its equivalent prior to ICU admission.
