# Supplementary material for: Plasma Neutrophil Elastase and Elafin Imbalance Is Associated with Acute Respiratory Distress Syndrome (ARDS) Development
Source: PLoS One. 2009 Feb 6;4(2):e4380. doi: 10.1371/journal.pone.0004380 (PMC2633615; doi:10.1371/journal.pone.0004380)
Supplement: Table S3 — Correlation coefficients (p value) among plasma profiles and APACHE III score (0.07 MB DOC) [file pone.0004380.s005.doc]

**Table S3. Correlation coefficients (*p* value) among plasma profiles and APACHE III score*^a^***

|  | **PI3** | **SLPI** | **HNE** | **HNE/PI3** | **APACHE** |
| --- | --- | --- | --- | --- | --- |
| **Control*^b^*** |  |  |  |  |  |
| **PI3** | - | **0.496**  **(<0.0001)** | 0.030  (0.815) | **-0.756**  **(<0.0001)** | **0.495**  **(<0.0001)** |
| **SLPI** |  | - | 0.078  (0.542) | **-0.334**  **(0.008)** | **0.509**  **(<0.0001)** |
| **HNE** |  |  | - | **0.632**  **(<0.0001)** | 0.172  (0.177) |
| **HNE/PI3** |  |  |  | - | **-0.271**  **(0.032)** |
| **APACHE** |  |  |  |  | - |
| **ARDS*^c^*** |  |  |  |  |  |
| **PI3** | - | **0.367**  **(0.004)** | **0.420**  **(0.0009)** | **-0.709**  **(<0.0001)** | **0.505**  **(<0.0001)** |
| **SLPI** |  | - | 0.096  (0.471) | **-0.305**  **(0.019)** | **0.339**  **(0.009)** |
| **HNE** |  |  | - | **0.343**  **(0.008)** | **0.506**  **(<0.0001)** |
| **HNE/PI3** |  |  |  | - | -0.130  (0.327) |
| **APACHE** |  |  |  |  | - |
| **Reference** |  |  |  |  |  |
| **PI3** | - | 0.139  (0.480) | -0.044  (0.824) | **-0.881**  **(<0.0001)** | NA |
| **SLPI** |  | - | 0.185  (0.346) | -0.032  (0.872 | NA |
| **HNE** |  |  | - | **0.512**  **(0.005)** | NA |
| **HNE/PI3** |  |  |  | - | NA |
| **APACHE** |  |  |  |  | NA |

ARDS, acute respiratory distress syndrome; Control, at-risk ICU control; PI3, neutrophil elastase inhibitor (elafin); SLPI, secretory leukocyte proteinase inhibitor; HNE, neutrophil elastase; APACHE, Acute Physiology and Chronic Health Evaluation III score.

1. APACHE III scores and log transformed plasma profiles had normal distributions in ARDS, control, and healthy reference, thus, Pearson correlation coefficients were calculated.
2. In controls, plasma profiles and APACHE III score were collected within 48-hour of ICU admission.
3. In ARDS, APACHE III score were collected within 48-hour of ICU admission, and plasma profiles were collected within 48-hour of ARDS diagnosis.
